# CRISPR-Cas9 strategies to insert MS2 stem-loops into endogenous loci in *Drosophila* embryos

**DOI:** 10.1016/j.xpro.2021.100380

**Published:** 2021-03-18

**Authors:** Caroline Hoppe, Hilary L. Ashe

**Affiliations:** 1 Faculty of Biology, Medicine and Health, University of Manchester, Manchester M13 9PT, UK

**Keywords:** Genetics, Model organisms, Molecular biology, CRISPR

## Abstract

CRISPR-Cas9 genome editing has transformed biology by enabling site-specific genome modifications to be simply engineered. Here, we describe two CRISPR-Cas9 approaches to introduce MS2 stem-loop sequences into endogenous gene loci in *Drosophila.* This can facilitate live imaging of nascent transcription in *Drosophila*.

For complete details on the use and execution of this protocol, please refer to Hoppe et al. (2020).

## Highlights

• CRISPR-Cas9 editing allows insertion of MS2 sequences into the *Drosophila* genome
• A one-step approach enables rapid insertion of MS2 loops or other sequences
• An alternative two-step protocol facilitates repeated targeting of the same locus

## Graphical abstract

**Figure:**
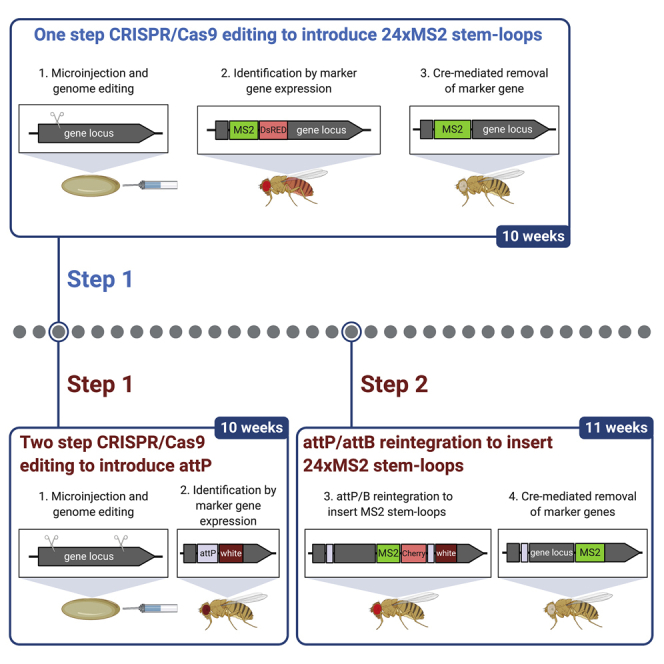

## Before you begin

### Experimental design considerations

**Figure 1:**
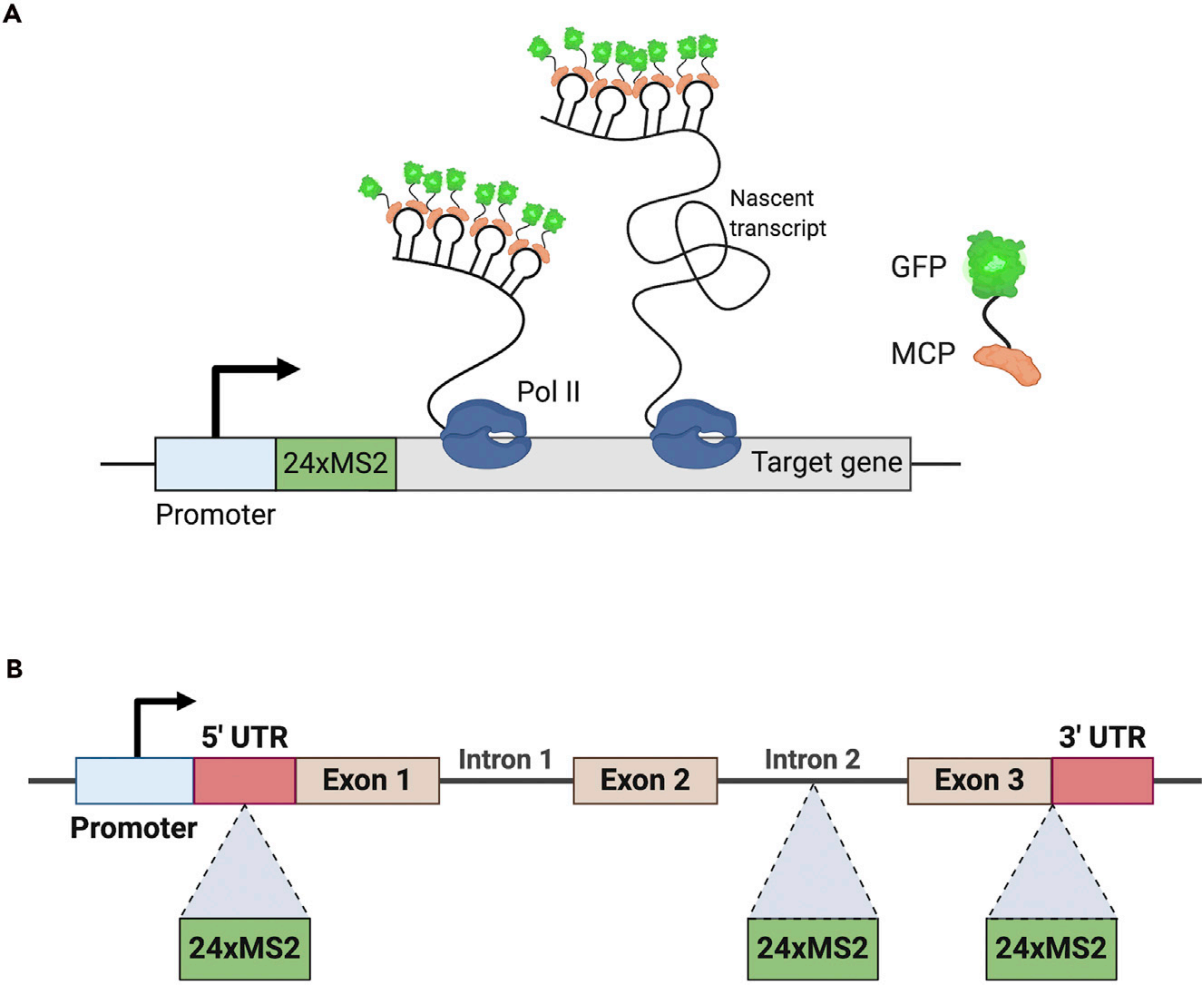
Inserting MS2 stem-loop sequences into an endogenous locus (A) The MS2/MCP live imaging system visualizes transcription using RNA stem-loop repeats that are bound by the MS2-coat protein (MCP) fused to a fluorescent protein. (B) MS2 stem-loops can be inserted into different endogenous genomic regions including 5′ and 3′ UTRs and intronic regions.

CRISPR-Cas9 genome editing uses a guide RNA (gRNA) to target the Cas9 endonuclease protein to the region of interest in the genome, where it introduces a site-specific double-strand break in the DNA (Adli, 2018; Doudna and Charpentier, 2014; Hsu et al., 2014). Homology-directed repair (HDR) can be used in conjunction with a DNA template to repair Cas9 induced dsDNA breaks and achieve precise genome editing. This method is used to introduce specific mutations or to insert exogenous DNA sequences into a genome (Adli, 2018; Doudna and Charpentier, 2014; Hsu et al., 2014). Here, we describe CRISPR-Cas9 protocols to introduce MS2 sequences into a gene in *Drosophila*, so that live imaging of transcription can subsequently be performed using the MS2/MS2 coat protein (MCP) system (Figure 1A) (Pichon et al., 2018). While some *Drosophila* studies have used short reporter transgenes carrying MS2 sequences (Falo-Sanjuan et al., 2019; Fukaya et al., 2017; Garcia et al., 2013; Lucas et al., 2013), inserting MS2 sequences into endogenous gene loci using CRISPR will allow transcription dynamics to be more accurately captured by ensuring all regulatory sequences are present.

One experimental consideration is how many times it may be useful to target the same gene region. If the goal is simply to introduce 24 copies of the MS2 stem-loop sequences, then a one-step CRISPR approach, which directly inserts the MS2 cassette, will be quicker and simpler. Moreover, CRISPR-Cas9 events inserting a sequence into the genome directly, based on a single cut, occur more frequently than the replacement of sequences that include large deletions (Poernbacher et al., 2019). Alternatively, a two-step approach can be used when it is advantageous to repeatedly target the same gene locus. This approach deletes sequences from the gene locus and concomitantly inserts an attP site, which subsequently allows easy targeting of the region, through ΦC31-mediated reintegration, to add back different versions of the deleted sequence with MS2 stem-loops. For example, deletion of sequences from upstream of the promoter where enhancer(s) are located to within the 5′ UTR will allow different mutations to be subsequently introduced into enhancer sequences or the core promoter.

Another consideration is which MS2 stem-loops to use, as different versions are available. The commonly used loops in flies, 24XMS2SL-stable, have an extremely high affinity for the coat protein, which aids visualization. However, the strong RNA-protein interaction has been suggested to prevent RNA degradation and produce decay fragments in yeast (Garcia and Parker, 2015). Improved versions of the MS2 loops include less repetitive stem-loop arrays (MBSV5; Addgene, Cat# 84561) (Wu et al., 2015), allowing for PCR amplification and sequencing, and stem-loops that have reduced affinity for the coat protein with different linker lengths (MBSV6 and MBSV7; Addgene, Cat# 104391 and 140705) (Tutucci et al., 2018). The fluorescent signal can be increased by introducing a greater number of stem-loops into a locus as shown for a 128xMS2 cassette (Tantale et al., 2016). Additionally, there is an orthogonal PP7 stem-loop system, with the PP7 coat protein (Chao et al., 2008; Pichon et al., 2018), which can be used with MS2 or as an alternative to it. The two-step CRISPR protocol will facilitate the testing of different numbers of MS2 loops, for example, or insertion of PP7 sequences to allow simultaneous imaging of both alleles of a gene.

Finally, the MS2 stem-loops can be inserted into different genomic locations, such as the 5′ UTR, 3′ UTR or intronic regions, with advantages and disadvantages to each (Ferraro et al., 2016) (Figure 1B). The fluorescent signal will be brighter when the stem-loops are positioned in the 5′ UTR compared to the 3′ UTR, as the loops in the 5′ UTR will be transcribed earlier and be available for MCP binding for longer (Garcia et al., 2013). Inserting loops in the 5′ UTR can potentially stall the scanning 40S ribosomal subunit and inhibit translation (Palam et al., 2011; Vattem and Wek, 2004), whereas introducing the loops into the 3′ UTR may disrupt mRNA regulation (Mayr, 2017). While neither of these is likely to alter the transcription dynamics, the fly stocks with the MS2 insertion may show reduced viability. Insertion of stem-loops into a large intron is not predicted to greatly affect splicing, although if splicing is efficient the loops will be very short lived decreasing the fluorescent signal.

***Note:*** For a protocol outlining how to image and quantitate transcription in embryos based on the MS2 system, please refer to . Hoppe and Ashe (2021)

### CRISPR design outline

**Timing: one-step CRISPR 10 weeks total**

**Timing: two-step CRISPR 10 weeks for CRISPR-Cas9 mediated targeting of an endogenous locus and 11 weeks total to reintegrate MS2 stem-loops into the endogenous locus**

**Figure 2:**
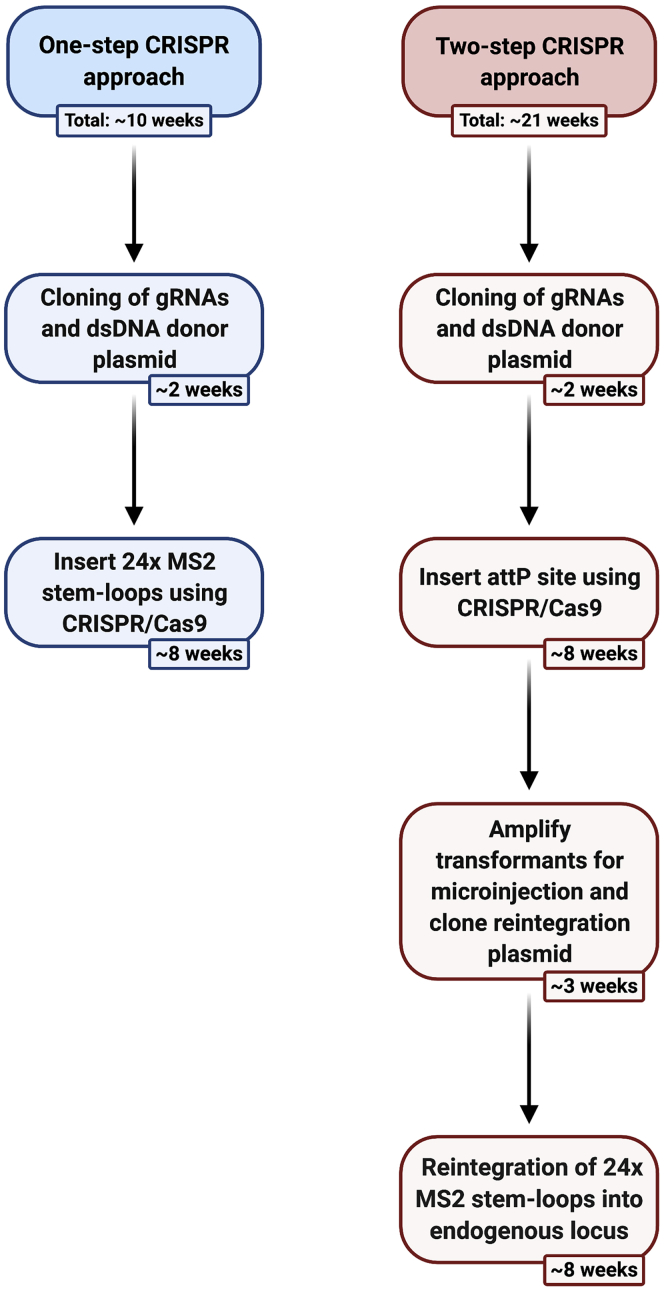
Overview showing the genome editing approaches covered in this protocol The major steps of the two CRISPR-Cas9 approaches are outlined with associated timings.

We outline two different CRISPR approaches, a one-step method using one cleavage site and a two-step approach that uses two DNA cleavage sites (Figure 2). While MS2 sequences are used as an example here, other sequences can be inserted using the methods described.

**Figure 3:**
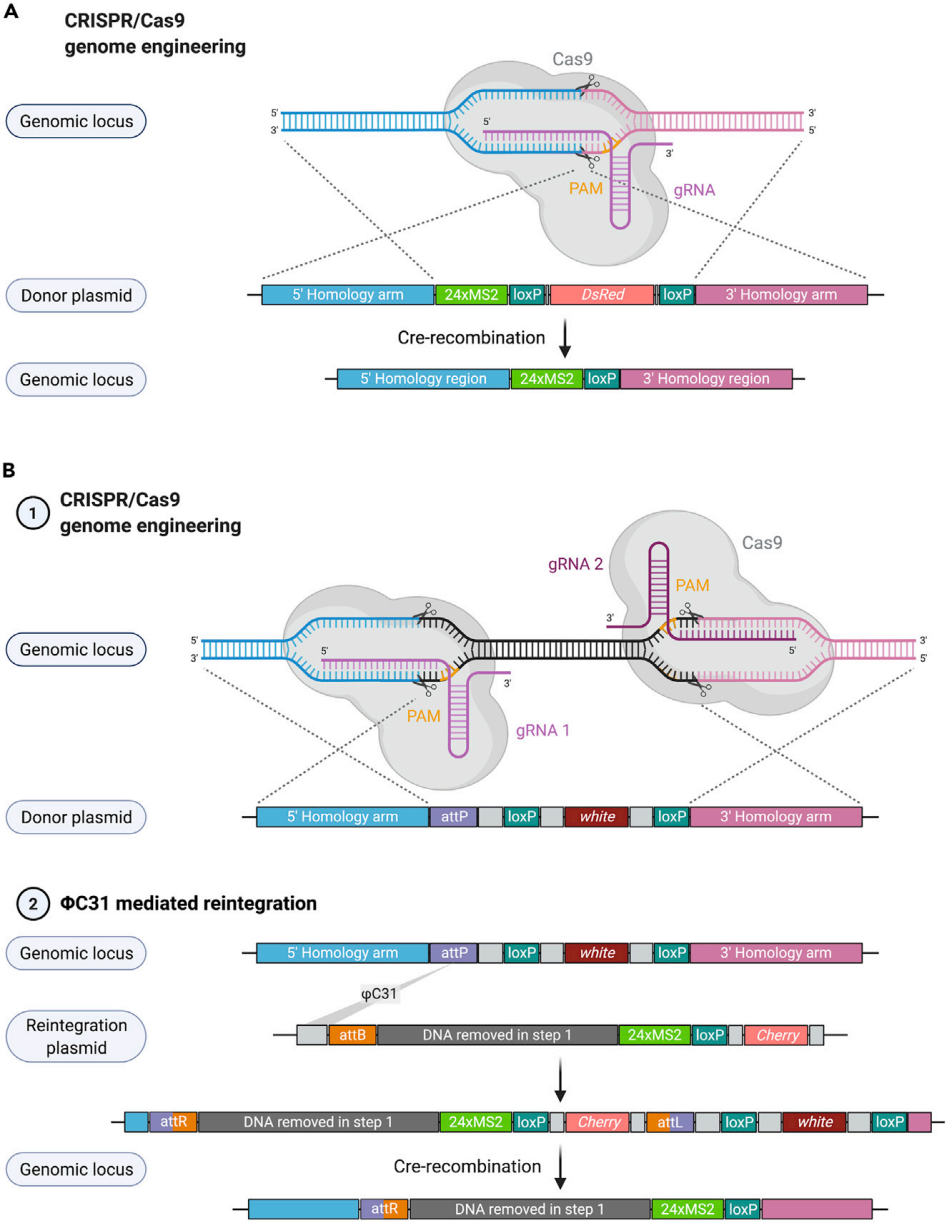
Overview of CRISPR-Cas9 genome engineering approaches (A) The one-step CRISPR approach uses a single site for gRNA directed, Cas9 mediated cleavage. A dsDNA donor plasmid is used for HDR and inserts 24xMS2 stem-loops into the endogenous gene locus. The donor plasmid also contains loxP sites and a marker gene. (B) For the two-step CRISPR approach, the genome is cut at two cleavage sites by gRNA directed, Cas9 mediated cleavage. The dsDNA donor plasmid contains loxP sites, an attP recombination site and a marker gene (1). In the second step, the previously removed DNA sequence together with MS2 stem-loops is reintegrated into the genome using attP/B recombination and the combined marker cassette is subsequently removed by Cre recombination between the two outermost LoxP sites (2).

In both approaches, CRISPR-Cas9-mediated HDR is performed using a dsDNA donor plasmid. The donor contains a gene encoding a visible transformation marker (for example *DsRed* or *mini-white*) flanked by loxP sites for Cre-recombinase-mediated removal and a multiple cloning site (MCS) for homology arm (HA) insertion (Figures 3A and 3B). The donor plasmid for the two-step CRISPR approach additionally contains an attP ΦC31 phage recombination site (Figure 3B), which can be used to reintegrate specific sequences including MS2 stem-loops in the second step.

In short, the one-step CRISPR strategy uses a single cut site to introduce MS2 stem-loop sequences directly adjacent to the cleavage site into the endogenous locus together with a selectable marker that is subsequently removed (Figure 3A) (Fukaya, 2020; Gratz et al., 2015; Lim et al., 2018b, 2018a).

The two-step CRISPR approach uses two cut sites to first insert an attP ΦC31 phage recombination site into the gene region of interest. In the second step, the attP ΦC31 recombination site is used to integrate MS2 stem-loops and the DNA sequences that were initially removed in step 1 (Figure 3B) (Baena-Lopez et al., 2013; Hoppe et al., 2020).

***Note:*** If the protocols described here are to be used to insert a protein tag, the exact position of the tag can be chosen by removing a region from the genome using two gRNAs, whereas a single cut site will place the tag sequences directly adjacent to the cut site and therefore be limited by gRNA availability. The chances for off-target effects are increased when using two gRNAs versus one.

***Note:*** Different CRISPR-Cas9 approaches haven been documented in *Drosophila* and mainly differ in the delivery of gRNAs and Cas9. These components can be injected in the form of expression plasmids or *in vitro* transcribed RNA together with the donor plasmid (Bassett et al., 2013; Gratz et al., 2013; Yu et al., 2013). Expressing *Cas9* (Gratz et al., 2014; Ren et al., 2013) or *Cas9* and gRNAs (Kondo et al., 2013; Port et al., 2014) stably as a transgene(s) was shown to result in increased CRISPR efficiency and consistency. The efficiency of different Cas9 transgenes and gRNA expression systems have been compared by Port et al. (2014, 2015). A summary of CRISPR-Cas9 approaches, such as generation of loss-of-function alleles or complex gene modifications and their experimental considerations can be found in Gratz et al. (2015). Experimental designs to insert tags into coding sequences through one-step or two-step CRISPR-Cas9 approaches are summarized by Poernbacher et al. (2019).

***Alternatives:*** Alternative HDR mediated CRISPR-Cas9 genome editing techniques can be used to avoid genomic scarring. In *Drosophila*, scarless strategies have been developed that first introduce a marker gene, which is replaced, in a second step, by the desired sequences (Kanca et al., 2019; Lamb et al., 2017). Another scarless approach uses the pHD-ScarlessDsRed donor plasmid and requires the presence of a genomic TTAA site in close proximity to the target region. The TTAA site is targeted by a PiggyBac transposon to introduce a screenable marker (http://flycrispr.molbio.wisc.edu/scarless).

### Target site identification

**Timing: 2 h**

This section describes the identification of DNA cleavage sites and the design of gRNAs. gRNAs are generated to direct the Cas9 endonuclease to genomic regions of interest to introduce double-stranded DNA breaks. The gRNA is directed to the genomic region through base pairing with the 20 nucleotide (nt) genomic target sequence (Figures 3A and 3B).

#### One-step CRISPR

1. Identify potential target sequences for DNA cleavage within the genomic region of interest by using the publicly available flyCRISPR target finder (https://flycrispr.org/target-finder/).
  a. Enter the genomic DNA sequence to find CRISPR target sites and, where appropriate, select the reference genome based on the *Drosophila Cas9* line that will be used.
  b. Optimal target sequences are 20 nt in length and located next to a 3 nt protospacer adjacent motif (PAM), which is required for DNA cleavage (Jinek et al., 2012).
  c. Select CRISPR targets with a 5′ G for optimized U6-driven transcription or add a 5′ G during the oligonucleotide design.
  d. On the next page select high stringency. To reduce the probability for off-target effects the option “NGG PAM sites only” can be selected.
  e. The website suggests cleavage target sites and highlights possible off-target sites. Carefully choose the target site best suited for the experiment (see Notes).
2. Select “design experiment” to view oligonucleotide sequences for the selected target. Order two oligonucleotides suggested by the website to generate one gRNA.
  a. Oligonucleotides contain CTTC 5′ and CAAA 3′ overhangs respectively, which are compatible with overhangs obtained in the pU6-BbsI-gRNA plasmid after BbsI digestion.
  b. Oligonucleotides should be ordered with the 5′ end phosphorylated or should be phosphorylated using T4 polynucleotide kinase before use.

#### Two-step CRISPR

3. Identify one target sequence for DNA cleavage on either side of the genomic region of interest and follow the steps outlined above.

***Note:*** If the target site search returned no results, consider lower stringency, shorter or alternative target sequences or selecting targets with possible off-target sites (see troubleshooting 1).

***Note:*** The DNA cleavage site is located 3 nt away from the PAM sequence within the target sequence (Figures 3A and 3B).

***Note:*** Some genomic regions exhibit reduced CRISPR efficiency. The reasons for locus-specific effects on CRISPR efficiency are poorly understood. The cleavage efficiency for *Drosophila* regions can be predicted using an online tool developed by the Perrimon group (Harvard Medical School). This prediction tool uses data from high throughput experiments in S2 cells (https://www.flyrnai.org/evaluateCrispr/).

**CRITICAL:** When selecting gRNAs, avoid those recognizing sequences overlapping or close to known regulatory motifs in the DNA or mRNA such as enhancers, binding sites for transcription factors or RNA binding proteins, or splice sites. The integration of DNA sequences at these sites may disrupt their regulatory function, as the methods described leave a small scar in the genome.

**CRITICAL:** Single nucleotide polymorphisms (SNPs) can occur naturally and could lower CRISPR efficiency when present in the targeting sequence. To verify that no SNP is present in the target sequences or PAM sites, sequence the genomic region in the lab stock of the fly line that will be edited before cloning gRNAs or check the genome sequence if available.

### Homology arm design

**Timing: 2 h**

This section describes the design of HAs that contain upstream/downstream genomic regions bordering the target cleavage site. HAs will be inserted into a dsDNA donor plasmid for HDR (see description in the step-by-step method details section).

**CRITICAL:** Check the sequence of the genomic regions around the target sites in the fly line chosen for editing before cloning, either using the genome sequence if available or by sequencing the specific regions.

***Note:*** A homology length of ∼1 kb was found to be efficient for integration. The efficiency of different homology length (100 bp to 7.5 kb) in dsDNA donors was investigated in detail by Beumer et al. (2013) and Kanca et al. (2019).

#### One-step CRISPR

4. Design the HAs to be approximately 1 kb in length and directly adjacent to the genomic cleavage site for efficient HDR.
5. Design primers to amplify HAs from genomic DNA.
  a. To the primer ends add restriction enzyme target sites that are present in the multiple cloning site (MCS) in the donor plasmid but absent from the HAs (for details see Figure 5).

***Note:*** Under certain circumstances the PAM sequence needs to be mutated, for example if a one-step CRISPR approach with two guide RNAs is chosen, as if the endogenous genomic DNA region is inserted directly next to the HA in the donor plasmid, the full guide and PAM sequences can be reformed. To prevent targeting of the donor plasmid by Cas9 and repeated targeting of the endogenous locus, a point mutation is necessary to mutate the “NGG” PAM (Gratz et al., 2015). In the example described here using one gRNA, the full target sequence will be disrupted by the MS2 stem-loop insertion at the cut site and therefore, no point mutation is necessary.

#### Two-step CRISPR

6. Design HAs to be approximately 1 kb in length. If gRNA/PAM sequences are in the PAM-in configuration as highlighted in this example (Figure 3B), HAs should not contain the 6 bps between the cleavage site and the end of the PAM sequence. Inclusion of the full target sequence will make the donor plasmid a target for Cas9 cleavage (Gratz et al., 2015).
7. Design primers to amplify the HAs from genomic DNA.
  a. Add restriction enzyme target sites, which are present in the donor plasmid multi-cloning sites (MCS) but absent from the HAs, to the primer ends (for details see Figure 6).

***Alternatives:*** If the restriction sites to be used are present in the HA sequences, use In-Fusion or Gibson cloning, or commercially synthesize the plasmid DNA.

### Design reintegration fragment

**Timing: 2 h**

The second step of the two-step CRISPR engineering approach uses ϕC31 integrase-mediated, site-specific transgenesis to introduce the MS2 stem-loops and the genomic region, which was removed as part of step 1, back into the genome (Figure 3B). This section outlines the primer design to generate inserts and the cloning procedure will be outlined in the step-by-step method details section.

#### Two-step CRISPR

8. Design primers to amplify the genomic region, which was removed in CRISPR step 1. In this example, the genomic DNA region will be inserted upstream of MS2 stem-loops into the RIV^cherry^+24xMS2-stem-loop plasmid (available upon request) using EcoRI and NotI restriction sites respectively (for details see Figure 9). Use different restriction sites, if these sites are present in the genomic region used for reintegration.
9. Order PCR primers to amplify the genomic region, containing sequences to insert an EcoRI (forward primer) and NotI (reverse primer) restriction site.

***Alternatives:*** Instead of a RIV^cherry^ backbone, the RIV^white^ plasmid can be used which contains a *mini-white* marker gene (Baena-Lopez et al., 2013). In order to use this plasmid backbone, the *mini-white* marker, inserted by the HDR donor plasmid, needs to be removed by Cre-mediated recombination before reintegration or a donor plasmid with a different marker gene needs to be used for HDR.

***Alternatives:*** Instead of using classical cloning techniques to insert DNA sequences using restriction sites, other methods such as In-Fusion or Gibson cloning methods can be used to reduce the insertion of ectopic nucleotides into the endogenous genome. Alternatively, plasmids can be commercially synthesized that lack any additional nucleotides that would be inserted into the genome. Scarless CRISPR methods have also been described (Kanca et al., 2019; Lamb et al., 2017) but are generally more complicated, whereas the addition of a scar here is not viewed as particularly problematic given that additional MS2 sequences are being inserted.

## Key resources table

**Table:** 

REAGENT or RESOURCE	SOURCE	IDENTIFIER
**Chemicals, peptides, and recombinant proteins**		

BbsI-HF restriction enzyme	New England Biolabs	Cat# R3539
EcoRI-HF restriction enzyme	New England Biolabs	Cat# R3101
NotI-HF restriction enzyme	New England Biolabs	Cat# R3189
Calf intestinal alkaline phosphatase	New England Biolabs	Cat# M0290
T4 DNA ligase	New England Biolabs	Cat# M0202
Stellar competent cells	TaKaRa	Cat# 636763
Q5 high-fidelity DNA polymerase	New England Biolabs	Cat# M0491
Phusion DNA polymerases	Thermo Fisher Scientific	Cat# F530
Nuclease-free water	Thermo Fisher Scientific	Cat# AM9938

**Critical commercial assays**		

PureLink Quick Plasmid Miniprep Kit	Thermo Fisher Scientific	Cat# K210010
Maxi Prep Plus Kit	Qiagen	Cat# 12963
QIAquick PCR and Gel Cleanup Kit	Qiagen	Cat# 28506

**Experimental models: organisms/strains**		

*yw, nos-Cas9*	Ashe Lab	N/A
*D. melanogaster*; *y*^*1*^*w*^*67c23*^; *sna*^*Sco*^*/CyO*, P{w^*+mC*^*=Crew*}DH1	Bloomington Drosophila Stock Center	RRID:BDSC_1092
*y*^*1*^*M{RFP[3xP3.PB] GFP[E.3xP3]=vas-Cas9}ZH-2A w*^*1118*^*/FM7c*	Bloomington Drosophila Stock Center	RRID:BDSC_51323
*D. melanogaster*; *y*^*1*^*w∗* P{y^+t7.7^=nos-phiC31\int.NLS}X; *Dr*^*1*^*e*^*1*^*/TM3, Sb*^*1*^	Bloomington Drosophila Stock Center	RRID:BDSC_34771
*y*^*1*^*w*^*67c23*^	Bloomington Drosophila Stock Center	RRID:BDSC_6599
*y*^*1*^*w*^*67c23*^; *sna*^*Sco*^*/CyO*	Ashe Lab	N/A

**Oligonucleotides**		

Guide RNA oligonucleotides, 5′ phosphorylated	Sigma	N/A
T7 promoter, forward primer for sequence verification: TAATACGACTCACTATAGGG	Sigma	N/A
T3 promoter, forward primer for sequence verification: GCAATTAACCCTCACTAAAGG	Sigma	N/A

**Recombinant DNA**		

pCR4-24XMS2SL-stable	Bertrand et al., 1998	RRID: Addgene_31865
pUbC-FLAG-24xSuntagV4-oxEBFP-AID-baUTR1-24xMS2V5-Wpre	(Wu et al., 2016)	RRID: Addgene_84561
pET259-pUC57 24xMS2V6	Tutucci et al., 2018	RRID: Addgene_104391
pET263-pUC57 24xMS2V7	Tutucci et al., 2018	RRID: Addgene_140705
pBS-24xMS2-loxP-DsRed-loxP	Lim et al., 2018a	N/A
pTV^cherry^	Drosophila Genomics Resource Center Baena-Lopez et al., 2013	DGRC_1338
RIV^cherry^	Drosophila Genomics Resource Center Baena-Lopez et al., 2013	DGRC_ 1331
RIV^white^	Drosophila Genomics Resource Center Baena-Lopez et al., 2013	DGRC_ 1330
RIV^cherry^ + 24xMS2 stem-loops	This study	N/A
pU6-BbsI-chiRNA	Gratz et al., 2013	RRID:Addgene_45946
act-phiC31-integrase	Drosophila Genomics Resource Center	DGRC_1368

**Other**		

Millipore membrane filter, 0.22 μm pore size	Merck	Cat# SLGVR04NL

## Step-by-step method details

### gRNA oligonucleotide insertion into pU6-BbsI-gRNA plasmid

**Timing: ∼6 days**

**Figure 4:**
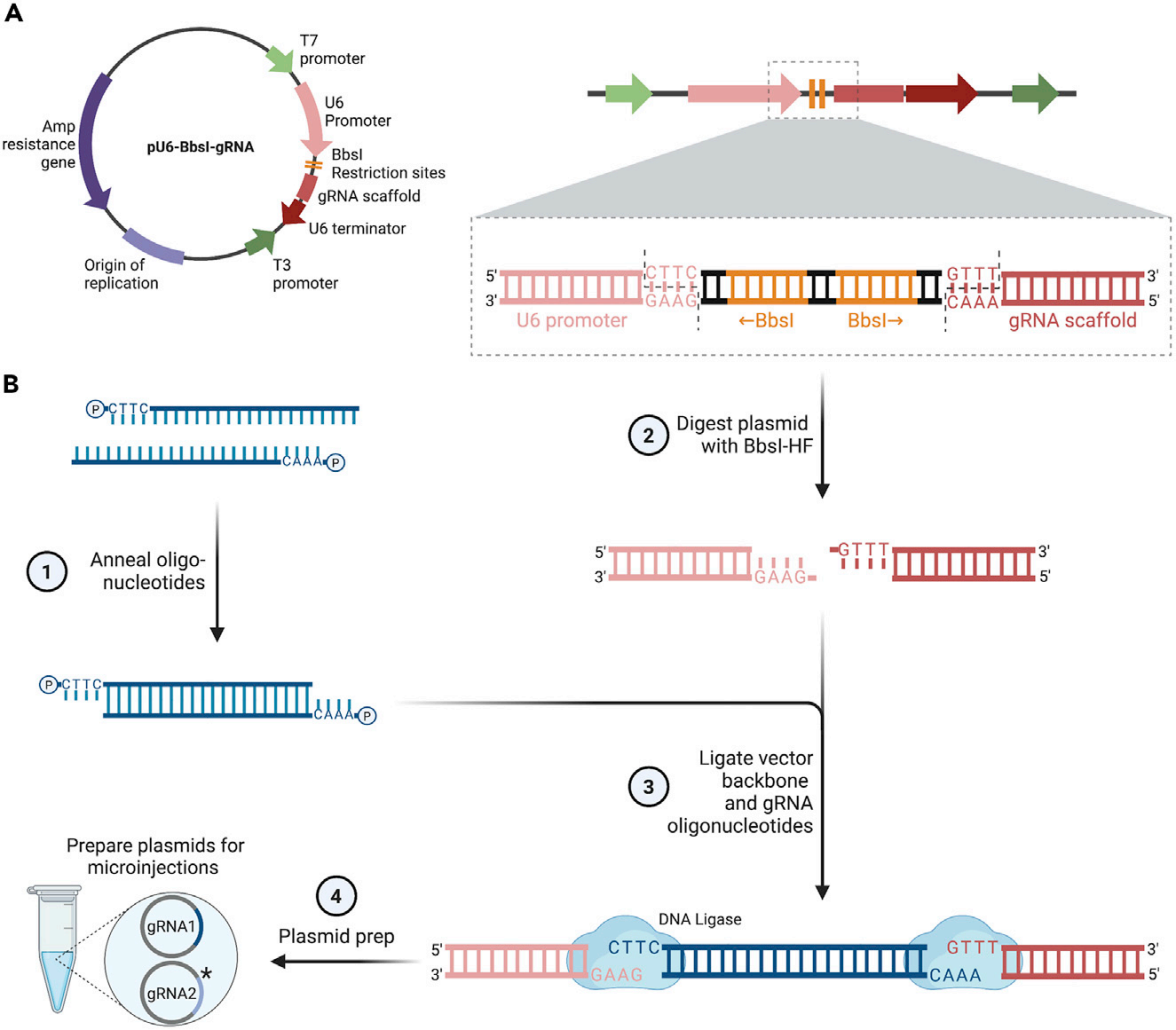
Cloning strategy to insert gRNAs into the pU6-BbsI-gRNA plasmid (A) The pU6-BbsI-gRNA plasmid is used to express gRNAs under the control of the U6 promoter in *Drosophila* embryos. (B) Oligonucleotides are annealed and overhangs allow insertion into the linearized vector (1). The vector is linearized by BbsI digestion (2) and the annealed oligonucleotides are ligated into the backbone (3). Plasmids with successfully inserted gRNA1 (one-step CRISPR) or gRNA1 and gRNA2 (two-step CRISPR) sequences are purified for microinjection (4). gRNA2 is labeled with an asterisk as it is only required for the two-step approach.

This section describes how to insert gRNA oligonucleotides into the pU6-BbsI-gRNA (Addgene, Cat# 45946) plasmid, which will be used to deliver gRNAs (Figure 4A). The gRNA target sequences start with a 5′ G, which allows for efficient U6 promoter-driven expression. Oligonucleotide sequences were determined in the before you begin section. This protocol was established by (Gratz et al., 2015) and is part of a cloning protocol selection by the FlyCRISPR website https://flycrispr.org/protocols/grna/.

#### One-step CRISPR and two-step CRISPR

1. Day 1. Reconstitute and anneal the designed phosphorylated oligonucleotides (Figure 4B).
  a. Set up the following annealing reaction for each oligonucleotide pair:

**Table:** 

Reagent	Amount	Final concentration
Oligonucleotide forward (100 μM)	1 μL	10 μM
Oligonucleotide reverse (100 μM)	1 μL	10 μM
T4 10× ligation buffer	1 μL	1×
ddH_2_O	7 μL	N/A
  b. Anneal oligonucleotides in a thermocycler at 95°C for 5 min, then ramp the temperature down to 25°C at a rate of −0.1°C/s (Figure 4B, step 1).

2. Digest vector for gRNA insertion with the BbsI-HF restriction enzyme, dephosphorylate and ligate the annealed gRNA oligonucleotides (Figure 4B).
  a. Digest 1 μg of pU6-BbsI-gRNA plasmid in a 40 μL reaction with 10 Units of BbsI-HF restriction enzyme for 2 h at 37°C (Figure 4B, step 2).
  b. Dephosphorylate the vector by adding Calf Intestinal Alkaline Phosphatase (NEB, Cat# M0290) to the reaction and incubate for an additional 30 min.
  c. Load digest reaction onto an 1% agarose gel and run at 100 V for about 30 min . Gel purify the digested plasmid to remove any undigested plasmid (for example QIAquick PCR & Gel Cleanup Kit; Qiagen, Cat# 28506).
  d. Ligate the annealed gRNA oligos into the linearized pU6-BbsI-gRNA vector using T4 DNA ligase (NEB, Cat# M0202) (Figure 4B, step 3).
  e. Transform ligation reactions into competent cells, for example Stellar competent cells (TaKaRa, Cat# 636763).
3. Confirm gRNA oligonucleotide insertion by sequencing minipreps (for example PureLink Quick Plasmid Miniprep Kit; Thermo Fisher Scientific, Cat# K210010) with T7 or T3 primers.
  a. Day 2. Pick bacterial colonies and set up liquid cultures to grow for approximately 14 h.
  b. Day 3. Perform the miniprep protocol according to the manufacturer’s instructions and send the DNA sample for sequencing.
  c. Day 5. Verify gRNA insertion based on DNA sequencing (see troubleshooting 2).
  d. Start liquid cultures and grow over night for approximately 14 h.
4. Day 6. Purification of plasmid DNA after successful verification of oligonucleotide insertion (Figure 4B).
  a. Use a kit to prepare plasmid DNA (for one gRNA for one-step CRISPR and two gRNAs for two-step CRISPR) that is sufficiently pure for microinjection (for example Maxi Prep Plus Kit; Qiagen, Cat# 12963) (Figure 4B, step 4).
    i. Generally, elute in nuclease-free water (Thermo Fisher Scientific, Cat# AM9938).
    ii. Use the extra wash step when suggested in the DNA prep kit protocol.
    iii. Filter eluted DNA using Millipore filters (Merck, Cat. # SLGVR04NL) and spin down prior to injections.

***Alternatives:***Both gRNAs can be expressed simultaneously from the pCFD4 or pCFD5 plasmids (http://www.crisprflydesign.org/plasmids/).

**Pause point:** The plasmids can be stored at −20°C for years.

### Introduction of homology arms into the dsDNA donor plasmid

**Timing: ∼11 days**

This section outlines how to generate a dsDNA donor plasmid that is used for HDR after Cas9 mediated cleavage.

**Figure 5:**
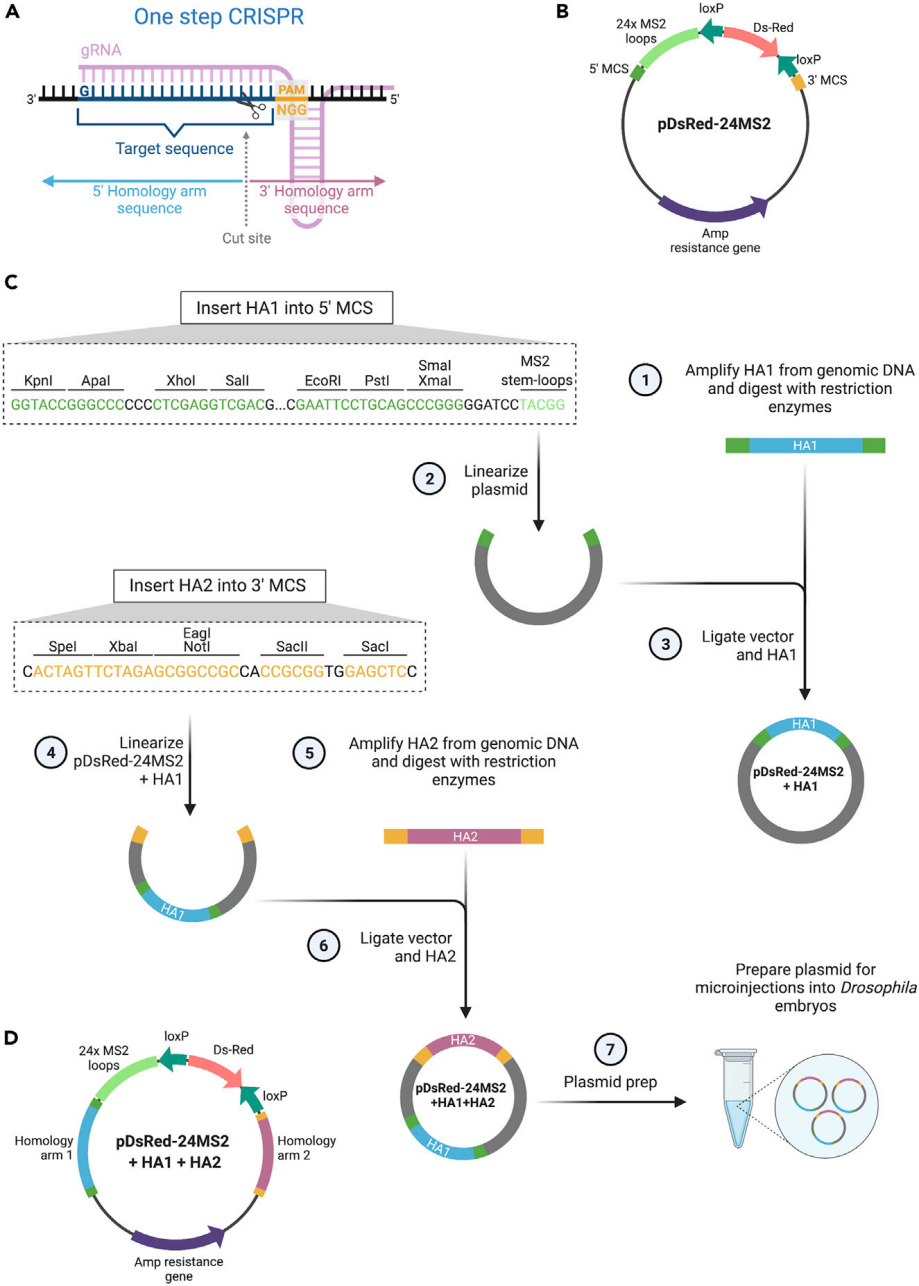
Cloning strategy to insert HAs into the pDsRed- 24MS2 plasmid for one-step CRISPR (A) Detailed view of the target sequence that starts with a 5′G and ends with the NGG PAM site and the gRNA. HAs sit on either site of the Cas9 cleavage site, which is located in the target sequence, 3 nt away from the PAM sequence. (B) The pDsRed-24MS2 plasmid contains two MCSs for HA insertion, the *DsRed* marker gene flanked by loxP sites and 24xMS2 stem-loops. (C) HA1 is PCR amplified from genomic DNA (1). The plasmid backbone is linearized using restriction sites in the 5′ MCS (2) and ligated with the HA1 fragment (3). The resulting pDsRed-24MS2+HA1 plasmid is linearized by restriction digest (4) and the HA2 fragment is amplified from genomic DNA (5). HA2 is ligated into the linearized vector (6) and purified for microinjection (7). (D) The finished plasmid pDsRed-24MS2+HA1+HA2 is used as a dsDNA donor for CRISPR engineering.

For the one-step CRISPR approach, HAs are located directly upstream and downstream of the cut site where the MS2 loops will be inserted (Figure 5A). The donor plasmid contains a pBlueScript backbone, a *DsRed* maker gene placed between two loxP sites, 24xMS2 stem-loops and MCSs for HA insertion (Figure 5B) (Lim et al., 2018a). Other sequences can be inserted instead of 24xMS2 stem-loops.

**Figure 6:**
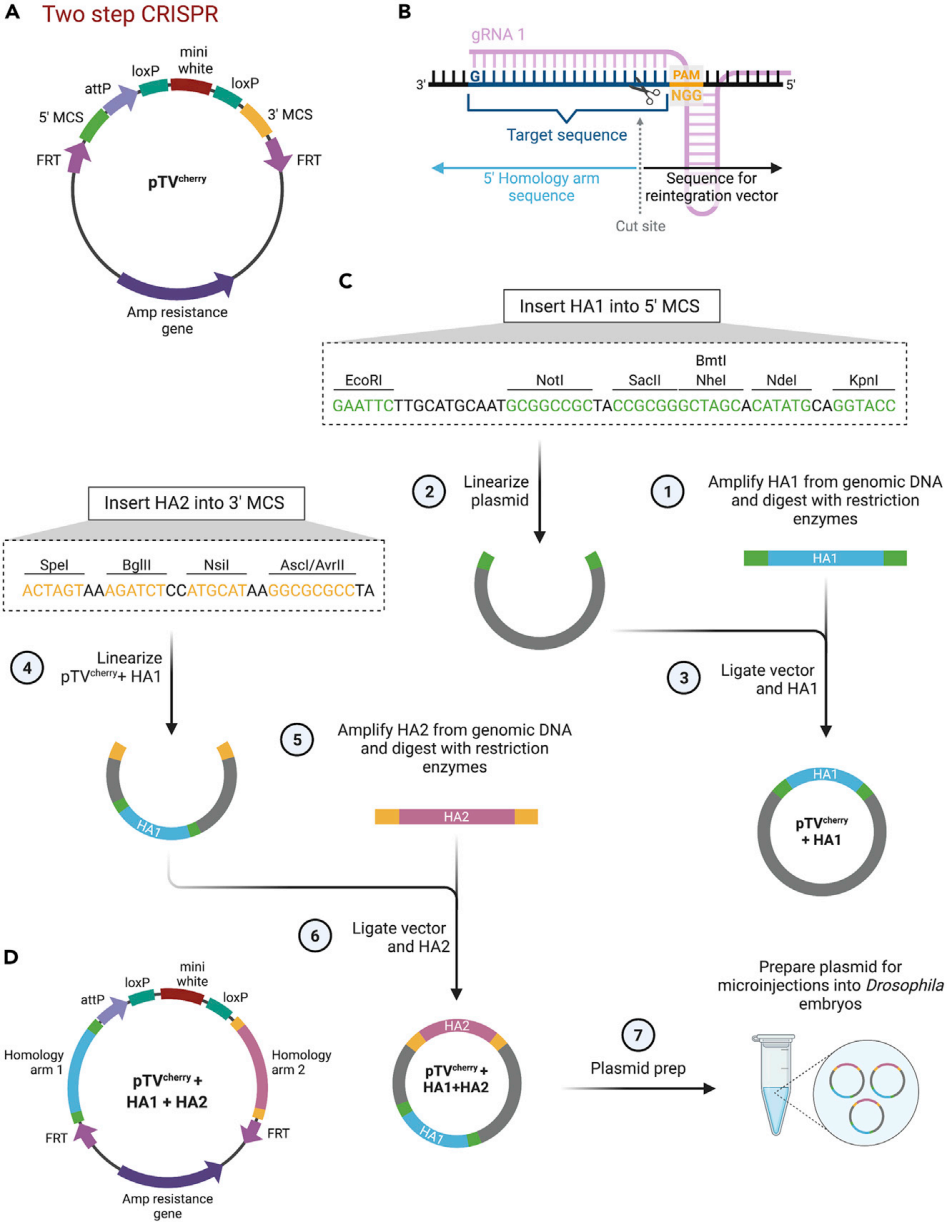
Cloning strategy to insert HAs into the pTV^cherry^ plasmid for two-step CRISPR (A) The pTV^cherry^ plasmid contains two MCSs to insert HA sequences for HDR, a *mini-white* marker gene, loxP and FRT sites, and an attP site. It also contains a *mCherry* gene (not shown). (B) Detailed view of the target sequence, starting with a 5′G and ending with the NGG PAM site. The Cas9 cleavage site is located 3 nt away from the PAM sequence and the 5′ HA ends upstream of the cleavage site. (C) HA1 is amplified from genomic DNA (1), the pTV^cherry^ plasmid is linearized using restriction sites in the 5′ MCS (2) and both are ligated (3). The pTV^cherry^+HA1 plasmid is linearized using restriction sites present in the 3′ MCS (4) and the HA2 insert is PCR amplified from genomic DNA (5) before it is ligated into the plasmid backbone (6). The plasmid containing both HAs is purified for microinjection (7). (D) The resulting pTV^cherry^+HA1+HA2 plasmid can be used as a dsDNA donor plasmid during CRISPR engineering.

For the two-step CRISPR approach, we use the pTV^Cherry^ plasmid (DGRC, Cat# 1338), which contains an attP reintegration site, a *mini-white* marker gene, loxP sites and is flanked by MCSs into which HA sequences are introduced (Figure 6A) (Baena-Lopez et al., 2013). The HAs are positioned upstream of the 5′ cut site (Figure 6B) and downstream of the 3′ cut site.

***Alternatives:*** Plasmids can be synthesized commercially.

***Note:*** The cloning to insert HAs into the dsDNA donor plasmid can be performed concurrently with the gRNA cloning to shorten the time needed to prepare plasmids for microinjection.

#### One-step CRISPR

5. Day 1. PCR amplify HA1 (5′) and HA2 (3′) from genomic DNA from the fly line that will be used for injection and digest with restriction enzymes (Figure 5C). Use primers that were designed in the before you begin section.
  a. Use a high-fidelity DNA polymerase, e.g., Q5 (NEB, Cat# M0491) or Phusion (Thermo Fisher Scientific, Cat# F530) for PCR (Figure 5C, step 1).
  b. Digest the purified PCR inserts, with the restriction enzymes whose recognition sequences were added to the HA primers, for insertion into the donor plasmid.
6. Insert HA1 into the linearized pBS-24xMS2-loxP-DsRed-loxP (hereafter referred to as pDsRed-24MS2) backbone plasmid (Figure 5C).
  a. Digest the donor plasmid backbone with the same restriction enzymes as the HA1 insert (Figure 5C, step 2).
  b. Dephosphorylate the vector with Calf Intestinal Alkaline Phosphatase.
  c. Gel purify the linearized vector.
  d. Ligate HA1 insert into the linear vector using T4 DNA ligase (Figure 5C, step 3).
  e. Transform the ligated plasmid into bacterial competent cells.
7. Days 2–5. Verify successful HA1 insertion by diagnostic restriction digests and sequencing.
  a. Day 2. Pick bacterial colonies and set up liquid cultures for approximately14 h.
  b. Day 3. Use liquid cultures to generate plasmid minipreps. Set up a diagnostic restriction digest https://www.addgene.org/protocols/diagnostic-digest/ using, for example, the restriction enzymes that were used to insert the HA1 insert. Check the DNA fragments using gel electrophoresis.
  c. Sequence minipreps, which show successful HA1 insertion by diagnostic digest, using primers positioned upstream and downstream of the insert.
  d. Day 5. Verify successful HA1 insertion from the sequencing reads (see troubleshooting 2).
8. Days 6–10. Clone the HA2 insert into the pDsRed-24MS2+HA1 plasmid (Figure 5C, steps 4–6).
  a. Repeat steps 5–7 amplifying and inserting HA2.
9. Day 11. Generate a high quality maxiprep (for example Maxi Prep Plus Kit; Qiagen, Cat# 12963, see additional considerations in step 4) of the donor pDsRed-24MS2+HA1+HA2 plasmid for microinjection (Figure 5C, step7). The finished plasmid is shown in Figure 5D.

***Note:*** To generate the pDsRed-24MS2 plasmid, the 24xMS2 stem-loops were extracted from the pCR4-24xMS2SL-stable plasmid (Addgene, Cat# 31865) by BglII and BamHI digestion and inserted into the BamHI restriction site of pBluescript together with two loxP sites and a *DsRed* marker gene (Lim et al., 2018a).

#### Two-step CRISPR

10. Cloning steps are similar to the one-step CRISPR approach outlined in detail above, but a different starting plasmid that contains an attP site is used (Figure 6). In short:
  a. Day 1: HA1 and 2 are amplified using a high-fidelity DNA polymerase and digested with restriction enzymes that recognisethe sequences that were added to the HA primers (Figure 6C, steps 1 and 5).
  b. HA1 is inserted into the linearized pTV^Cherry^ vector (Figure 6C, steps 2 and 3).
  c. Days 2–5. Verify successful HA1 insertion by diagnostic restriction digest and sequencing (see troubleshooting 2).
  d. Day 6. Ligate HA2 into the linearized pTV^Cherry^+HA1 plasmid (Figure 6C, steps 4 and 6).
  e. Days 7–10. Use diagnostic digests and sequencing to verify the successful insertion of HA2 in the plasmid.
11. Day 11. Generate a maxiprep of the pTV^Cherry^+HA1+HA2 plasmid for microinjection (see additional considerations in protocol step 4) (Figure 6C, step7). The finished pTV^Cherry^+HA1+HA2 plasmid is shown in Figure 6D.

**Pause point:** The plasmids can be stored at −20°C for years.

### Microinjection and selection of CRISPR edited flies

**Timing: One-step CRISPR ∼8 weeks; two-step CRISPR ∼8 weeks to insert attP and 3 weeks to amplify for microinjection**

#### One-step CRISPR and two-step CRISPR

**Figure 7:**
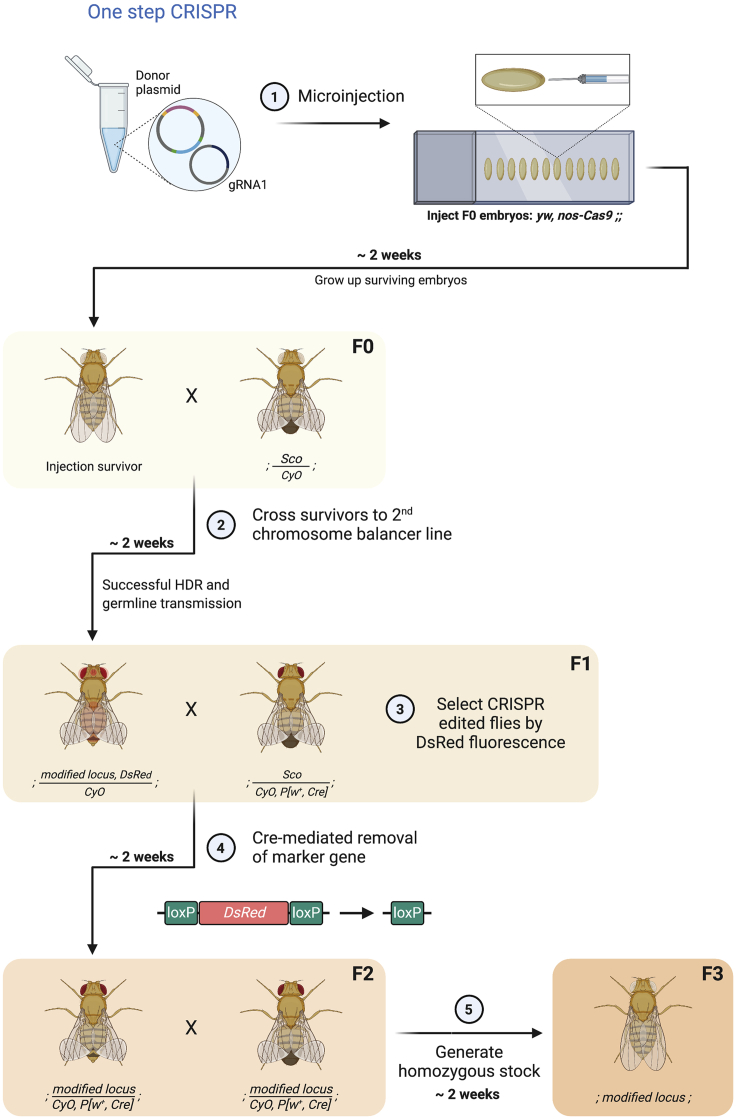
One-step CRISPR-Cas9 genome engineering in *Drosophila* embryos Together the pDsRed+24MS2+HA1+HA2 donor plasmid and the plasmid expressing gRNA1 are injected into *Drosophila* embryos of the genotype *yw, nos-Cas9* (F0) (1). Surviving flies (a female is shown as an example) are crossed to balancer flies (2). Putative CRISPR edited flies are identified by *DsRed* marker gene expression in F1 flies (3). Each CRISPR edited fly is individually crossed to *Cre*-recombinase expressing flies (4), resulting in the removal of the marker cassette from the genome (F2, select *w*^*+*^*CyO* flies and against *Sco*). Correct editing is verified by sequencing (not shown). Flies, now lacking the *DsRed* marker, are crossed to each other (5), resulting in F3 flies, homozygous for the modified gene locus. These flies can be used for live imaging experiments. In this crossing scheme all flies carry *yw* mutations on the X chromosome (not shown in the genotypes for simplicity).

**Figure 8:**
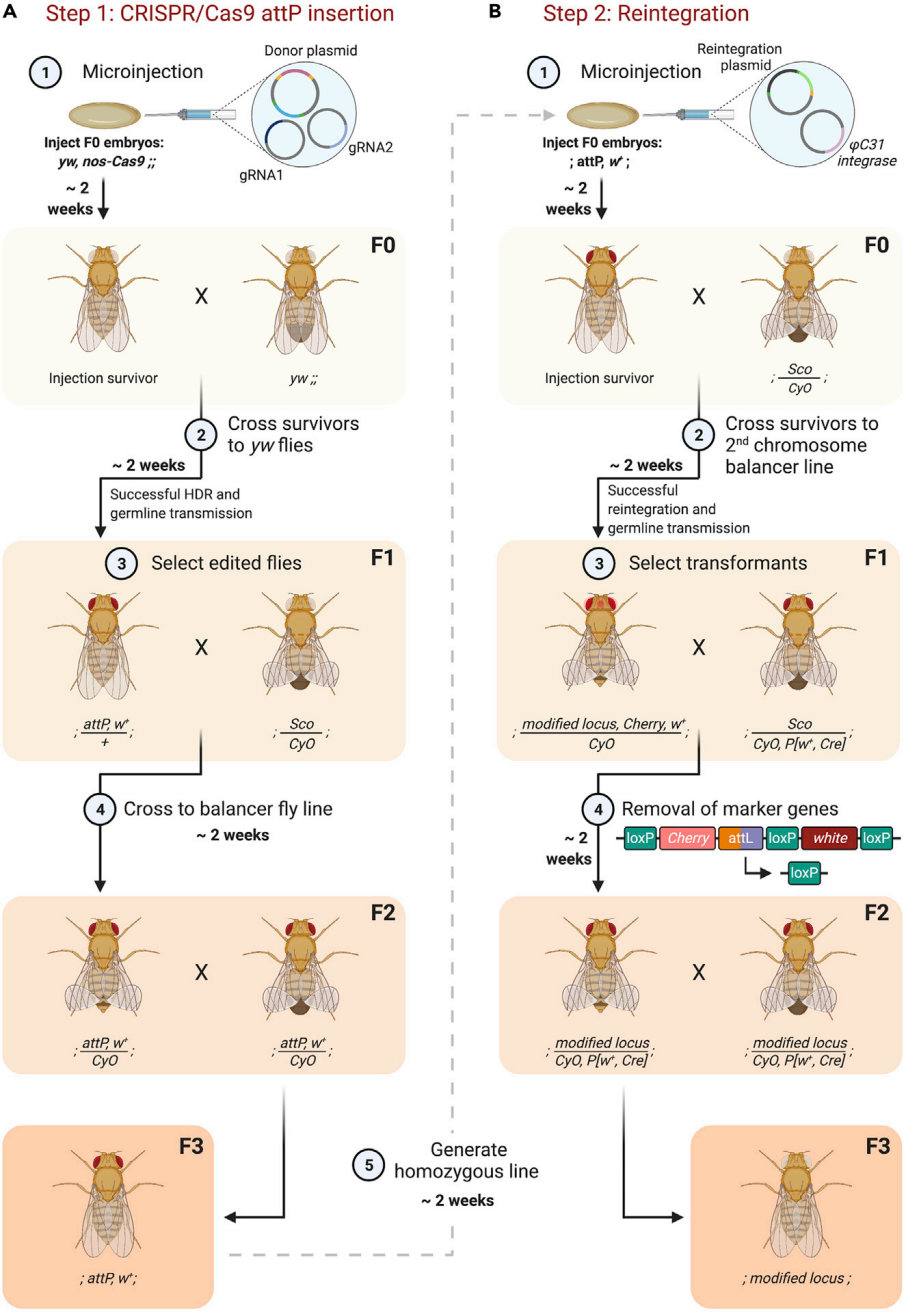
Two-step CRISPR-Cas9 genome engineering in *Drosophila* embryos and ϕC31 integrase mediated attP/attB recombination (A) The pTV^cherry^+HA1+HA2 plasmid together with gRNA1 and gRNA2 plasmids are injected into *Drosophila* embryos of the genotype *yw, nos-Cas9*;; (F0) (1). Survivors are crossed individually to *yw* flies (2). Flies that potentially have a correct editing event are identified by red eyes (*mini-white* marker) in the F1 generation (3). These flies are then crossed individually to a balancer fly line (4), before confirming the editing event by sequencing (not shown). Balanced flies from the single mating are then crossed to each other in the F2 generation (5) to obtain flies homozygous for the CRISPR inserted attP locus. (B) The reintegration plasmid, containing MS2 stem-loops, a *mCherry* marker gene and the genomic region removed during CRISPR-Cas9 genome engineering, and the plasmid expressing ϕC31 integrase are injected into *Drosophila* embryos carrying the attP site (F0) (1). Injected flies are crossed individually to balancer flies (2). Successful reintegration is detected by mCherry fluorescence in the eyes of F1 flies (3). Transformants are crossed to a *Cre*-recombinase expressing fly line to remove the combined marker region containing the *mini-white* and *mCherry* marker gene sequences between the two outermost loxP sites (4). F2 flies (select *w*^*+*^*CyO* flies and against *Sco*) that have lost mCherry fluorescence are crossed to each other to obtain flies homozygous for the modified gene locus (5). Note that the flies used in these injection and reintegration schemes also carry *yw* mutations on the X chromosome (not shown).

***Note:*** Initially inject 200–400 *Drosophila* embryos. More embryos can be injected if low recovery of edited flies is expected, for example due to targeting genomic regions on the X chromosome that may cause male lethality. Many commercial injection services are available that have optimized the injection protocol.

12. Week 1. The respective donor plasmid is injected together with one or two gRNA plasmids into *Cas9* expressing embryos. A list of *Cas9* expressing fly lines available can be found here: https://bdsc.indiana.edu/stocks/genome_editing/crispr_cas9.html.
  a. The examples chosen in Figures 7 and 8A (step 1) use the *yw, nos-Cas9* fly line (gift from Simon Collier and available from the Ashe lab).

**CRITICAL:** When using the *DsRed* marker gene or another fluorescent marker, make sure to use a *Cas9* transgenic line without fluorescent markers of similar wavelength. Here we use a line with *nos-Cas9* on the X- chromosome and no additional markers. This line has the advantage that it is compatible with CRISPR schemes based on selection with a fluorescent protein or *mini-white*, but relies on loss of *nos-Cas9* over time. In contrast, Cas9 lines marked with fluorescent proteins, such as *y*^*1*^*M{RFP[3xP3.PB] GFP[E.3xP3]=vas-Cas9}ZH-2A w*^*1118*^*/FM7c* [RRID:BDSC_51323], allow *Cas9* to be selected against in subsequent generations, but are incompatible with some donor plasmids.

***Note:*** The plasmid concentration to be injected varies. We inject 100 ng/μl of each gRNA plasmid and 500 ng/μl of the donor plasmid. Other concentrations have been tested (Gratz et al., 2015).

***Note:*** The examples in Figure 7 and 8A show the crossing schemes for a targeted gene locus on the 2^nd^ chromosome.

***Note:*** The example crossing scheme for the one-step CRISPR approach outlines a time efficient way to identify the CRISPR-Cas9 genome modification and remove the *Cre*-recombinase from the genome. Crossing the injection survivors to wildtype flies may increase the number of progeny, increasing the chances of finding a fly with a modified genome, but will then require the extra step of balancing the edited locus so it can be selected when the visible marker is removed by *Cre*.

#### One-step CRISPR

13. Week 3. Cross all surviving flies individually (F0 generation) to a 2^nd^ chromosome balancer fly line (Figure 7, step 2).
  a. The example in Figure 7, step 2, shows a surviving female.
  b. The balancer stock used in this example is *y*^1^ *w*^67c23^; *sna*^*Sco*^/*CyO* (Ashe lab).
14. Week 5. Identify flies that may have successful HDR and germline transmission by the presence of the *DsRed* marker in the F1 generation (see troubleshooting 3) (Figure 7, step 3).
  a. DsRed fluorescence is driven by a 3xP3 (*pax*) promoter and primarily expressed in the eyes and ocelli. Additionally, DsRed fluorescence can be detected in the abdomen.
15. Week 5. Cross the F1 CRISPR positive flies identified above individually to a *Cre*-recombinase expressing fly line to remove the *DsRed* marker gene flanked by loxP sites (Figure 7, step 4).
  a. Choose a *Cre* expressing fly line with a convenient balancer. A list of *Cre*-recombinase expressing fly lines can be found here: https://bdsc.indiana.edu/stocks/recombinases/recomb_alt.html.
  b. In Figure 7, step 4, a female F1 fly that has a modified gene locus over a *CyO* balancer is crossed to males expressing *Cre* recombinase on a *CyO* balancer [RRID:BDSC_1092].
    i. Set up individual crosses with a number of putative CRISPR positives in case of off-target effects.
    ii. After the cross has produced a sufficient amount of eggs and larvae are visible, verify the correct genome integration by purifying genomic DNA from the DsRed positive CRISPR edited fly and sequencing PCR amplified regions across the editing site troubleshooting 4.
    iii. Keep additional CRISPR positive flies as a backup.
16. Week 7. Identify flies that have undergone recombination (loss of *DsRed* expression) and carry the *CyO*, *P[w^+^*, *Cre]* expressing chromosome. Generate a homozygous stock by crossing the flies to each other (Figure 7, step 5).
17. End of week 8. In flies emerging from the cross, selecting flies homozygous for the insertion will allow the loss of *P[w^+^*, *Cre]*, located on the *CyO* balancer, from the genome. The homozygous flies allow the stock to be maintained and are ready to be used for live imaging experiments.

***Note:*** In the absence of selection, *nos-Cas9* will be lost from the genome over time. Other lines with *Cas9* linked to a fluorescent protein or *white* marker allow for it to be selected against in subsequent generations.

***Note:*** The one-step CRISPR approach will leave a loxP (34 bps) site and a few nucleotides (MCS) behind as a scar in the genome. The scarring can minimized by using restriction sites closest to the MS2 stem-loops/loxP in the pDsRed- 24MS2 plasmid.

#### Two-step CRISPR

18. Week 3. Cross all surviving flies to a *white*^*-*^ fly stock (F0 generation) in individual crosses (Figure 8A, step 2).
  a. The example in Figure 8A, step 2, shows a surviving female crossed to *y*^1^ *w*^67c23^ males [RRID:BDSC_6599].
19. Week 5. Identify F1 CRISPR edited flies by the presence of red eyes due to the *mini-white* marker gene (Figure 8A, step 3) (see troubleshooting 3). Cross single transformants to a 2^nd^ chromosome balancer fly line (for example *y*^1^ *w*^67c23^; *sna*^*Sco*^/*CyO* (Ashe lab)) (Figure 8A, step 4).
  a. Set up individual crosses with a number of potential CRISPR positives in case of off-target effects.
  b. After the cross has produced a sufficient amount of eggs and larvae are visible, verify correct genome integration by PCR and sequencing the targeted region in the adult fly troubleshooting 4.
20. Week 7. Select *w*^*+*^ *CyO* flies and cross them to each other to generate a stock that is homozygous for the attP insertion (lacking *CyO*) (Figure 8A, step 5). Alternatively, this will allow maintenance of a balanced stock if the CRISPR edit causes lethality (see Note section below).
21. Weeks 9–11. Amplify homozygous (or balanced where necessary) flies to obtain enough adults for a second round of microinjections.

***Note:*** attP containing flies will not be homozygous viable if the deleted genome region contains important regulatory elements such as promoters or enhancers. In this case the balanced stock can be used for injection of the reintegration plasmid.

***Note:*** The visible marker gene is not removed during this step, as the plasmid that will be used for reintegration through the attP site, contains a different marker. Both marker genes can be removed at the same time using Cre-recombinase after the reintegration step (Baena-Lopez et al., 2013).

### ϕC31 integrase-mediated site-specific transgenesis

**Timing: Cloning, 6 days; transgenesis, ∼8 weeks**

#### Two-step CRISPR

**Figure 9:**
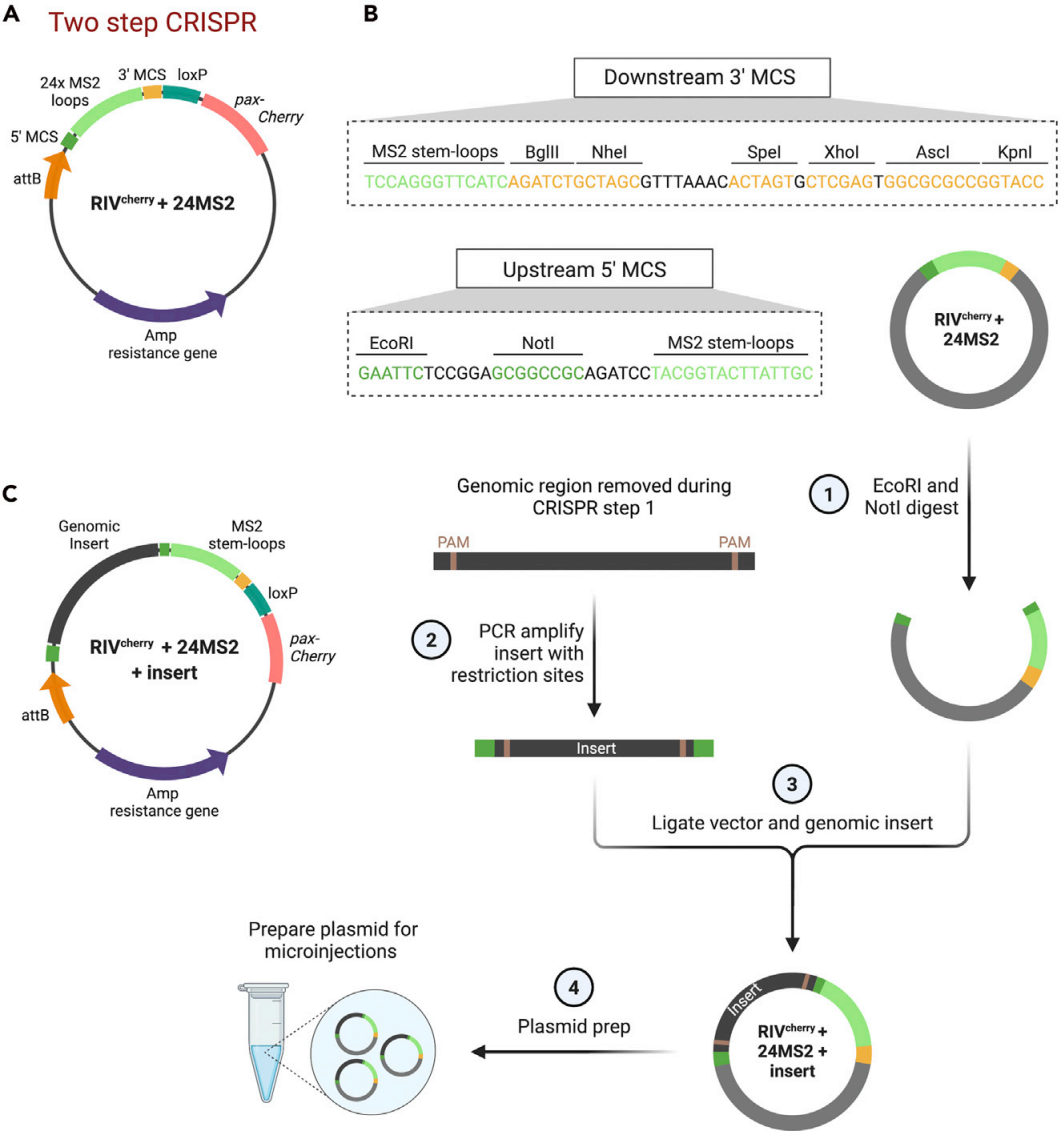
Cloning strategy for the reintegration plasmid used for two-step CRISPR (A) The RIV^cherry^+24xMS2-stem-loop plasmid contains an attB site for ϕC31 integrase-mediated recombination and the MS2 stem-loops. (B) The plasmid is linearized using EcoRI and NotI restriction sites that are part of the 5′ MCS (1). The genomic region, removed in the first CRISPR step, is amplified from genomic DNA and digested with EcoRI and NotI (2) followed by ligation into the linearized vector (3). After successful insertion, the plasmid is purified for microinjection (4). (C) The reintegration plasmid also contains a *pax-mCherry* marker gene to identify positives.

In this step, site-directed reintegration utilizing the attB-attP system is used to insert MS2 stem-loops and the sequences that were removed in the first CRISPR step into the endogenous gene locus. To this end, the previously deleted genomic sequences are inserted into the RIV^cherry^+24xMS2-stem-loop plasmid (Figure 9A), upstream of the MS2 stem-loops. Other sequences can be introduced into the genome instead of the 24xMS2 stem-loops by using a similar approach.

### Cloning

**Timing: 6 days**

***Note:*** It is most time efficient to perform the molecular cloning to insert the genomic region into the reintegration plasmid concurrently with the fly crosses described above that generate the homozygous CRISPR edited flies carrying an attP site.

***Note:*** The 24xMS2 stem-loops were extracted from the pCR4-24xMS2SL-stable plasmid (Addgene, Cat# 31865) by BglII and BamHI digestion and inserted into the BglII restriction site of RIV^cherry^ (DGRC, Cat# 1331) to generate the RIV^cherry^+24xMS2-stem-loop plasmid.

22. Day 1. Digest the RIV^cherry^+24xMS2-stem-loop plasmid with EcoRI and NotI restriction enzymes (Figure 9B, step 1). Cloning steps are very similar to cloning the HA sequences (outlined in detail above, see steps 5–9), so are summarized in this section (see troubleshooting 2).
23. PCR amplify the genomic region, which was removed during the first CRISPR step and sits inside of the HAs. This includes the cleavage site to PAM site sequence shown in black and orange, Figure 3B. Use primers designed in the before you begin section that insert an EcoRI and NotI restriction site at either end of the insert (Figure 9, step 2). Ligate the insert into the linearized plasmid (Figure 9B, step 3).
24. Days 3–5. Confirm the insertion by diagnostic digests and sequencing.
25. Day 6. Generate a maxiprep of the finished RIV^cherry^+24xMS2-stem-loop+genomic region plasmid (Figure 9C) for microinjection (Figure 9B, step 4) . Ensure the DNA is of high quality (see additional considerations in protocol step 4).

***Alternatives:*** The genomic DNA can be inserted downstream of the MS2 stem-loops using the 3′ MCS. If the MS2 stem-loops need to be placed within the genomic region that is reintegrated, two inserts can be generated and inserted upstream and downstream of the stem-loops respectively or alternative cloning strategies can be used.

### Transgenesis

**Timing: 8 weeks**

26. Week 1. Co-inject the RIV^cherry^+24xMS2-stem-loop+genomic region plasmid together with a ϕC31 integrase expression plasmid (act-phiC31-integrase, DGRC1368) into embryos from the homozygous attP CRISPR fly line amplified in step 21 (F0) (Figure 8B, step 1). ϕC31 integrase-mediated site-specific transgenesis allows for the recombination of attB and attP sites. The reintegration forms attL and attR sites and inserts the target DNA (Figure 3B, step 2).

***Alternatives:*** Instead of supplying a ϕC31 integrase plasmid, flies can first be crossed to a fly line that expresses ϕC31 integrase (for example RRID:BDSC_34771).

27. Week 3. Cross surviving F0 flies to 2^nd^ chromosome balancer flies (Figure 8B, step 2).
  a. The example in Figure 8B, step 2, shows a surviving female fly crossed to males of the *y*^1^ *w*^67c23^; *sna*^*Sco*^/*CyO* genotype (Ashe lab) .
  b. Individual crosses should be set up for a number of transformants in case of incorrect integration events.
28. Week 5. Select transformants based on *pax* promoter-driven mCherry fluorescence in the eyes of F1 flies (Figure 8B, step 3) (see troubleshooting 5).
29. Week 5. Remove the combined marker region, now containing 3 loxP sites, the *mini-white* marker and the *mCherry* marker by crossing F1 transformants to a *Cre*-recombinase expressing fly line (Figure 8B, step 4) (Baena-Lopez et al., 2013).
  a. Select an individual F1 female *CyO* fly that shows mCherry fluorescence and cross to males of the *CyO*, *P[w^+^*, *Cre*-*]* line BDSC_1092.
  b. After the cross has produced a sufficient amount of eggs and larvae are visible, sequence verify the correct integration of the MS2 stem-loops and associated sequences by sacrificing the adult transformant fly.
30. Week 7. To obtain flies ready for live imaging, the recombined flies (now lacking mCherry fluorescence) are crossed to each other to generate a homozygous stock.
  a. Cross females and males that carry the modified locus (are mCherry negative) and the *CyO* balancer (Figure 8B, step 5).
31. End of week 8. In the F3 generation select against the *CyO* balancer and the *white* marker (*P[w^+^*, *Cre])* to remove *Cre* from the genome. These flies are now homozygous for the genome modification, which allows for the stock to be maintained and it is ready to be used for live imaging experiments.

***Note:*** The two-step CRISPR approach will leave small scars in the genome in the form of the attR (attP/B) site at the 5′ end and one LoxP site (34 bps) at the 3′ end of the targeted locus. The plasmids used in this protocol contain minimal attP (50 bps) and attB (51 bps) sites (Baena-Lopez et al., 2013) that were established by Huang et al., (2009), reducing the attR sequence length contained in the final engineered fly line to 48 bps. The small attR size minimizes the amount of sequence to be added in addition to the MS2 stem-loop cassette, but adding additional sequences will be more problematic for other CRISPR applications that target the coding sequence. Additionally, a few nucleotides will have been inserted depending on the restriction sites that were used for cloning. Most of these additional nucleotides can be avoided by using In-Fusion or Gibson cloning or commercially synthesizing the plasmid.

## Expected outcomes

The expected outcome is the insertion of 24xMS2 stem-loops (or alternative donor sequence) into a specific position within the gene locus. Using the two-step CRISPR approach, the gene locus is marked with an attP site as an intermediate step, which can be used for further modification of the gene region. Tagging an endogenous locus with MS2 sequences will enable live imaging studies to investigate and quantitate nascent transcription dynamics. For our recent study investigating the transcriptional regulation of endogenous Bone Morphogenetic Protein target genes see Hoppe et al., 2020.

## Limitations

While inserting MS2 sequences into the genome using CRISPR gives the advantage of allowing the study of endogenous transcriptional activity, it is also time consuming. CRISPR modification takes more time than generating a transgene and inserting it into a targeted landing site within the genome. Therefore, the advantages of being able to investigate endogenous transcription dynamics versus studying a reporter transgene have to be balanced with the time it takes to generate the fly lines. In addition, once the attP line is made using the two-step approach, test sequences can be rapidly targeted to the gene locus.

Another limitation is that the addition of MS2 stem-loops could alter gene expression. Even though transcription is not expected to be altered, it is possible that the addition of MS2 stem-loops to the 5′ or 3′ UTR leads to changes in translation efficiency or mRNA regulation (Mayr, 2017; Palam et al., 2011; Vattem and Wek, 2004). These potential disruptions to gene expression could lead to a reduced viability of fly stocks.

Some regions of the genome appear resistant to targeting. However, as there is flexibility in the placement of the MS2 sequences and therefore the gRNAs that can be used, this is not a major issue here.

## Troubleshooting

### Problem 1

Target site search returned no results or only those with multiple off-target sites.

### Potential solution

If no target sites are found, consider lowering the stringency of the search. With a lower stringency, more target sites will be identified, as gRNAs often tolerate several mismatches in their seed sequence. The danger is that a lower stringency also increases the potential for off-target events.

The length of the seed sequence can be shortened to 16 nt to increase target site identification and reduce off-target probability. While sequences shorter than 16 nt can be used to guide Cas9, they are insufficient to promote endonuclease activity (Dahlman et al., 2015; Fu et al., 2014). It was shown that seed sequence lengths of 17 and 18 nt reduce the potential for off-target effects while at the same time retain normal efficiency in mammalian cells (Fu et al., 2014).

If no target sites are identified at the region of interest when using a single gRNA approach or only target sites with a high number of possible off-target binding sites are available, consider using two gRNAs. Choosing two target sites that are located upstream and downstream of the region of interest will allow modification of the region of interest but will require insertion of the DNA sequence between the cleavage sites into the donor plasmid to avoid a deletion.

Theoretically, other Cas proteins can be used that favor different PAM sequences and therefore make other target sites available. For example the Cas12a protein is available for use in *Drosophila* (Port et al., 2020).

Try to avoid off-target locations on the same chromosome arm or even the whole chromosome as the region of interest. Potential off-target events on other chromosomes can be crossed out.

### Problem 2

One of the plasmids cannot be generated.

### Potential solution

Generally, oligonucleotides are easy to clone so problems generating the plasmids to express the gRNAs seem unlikely. It may be more difficult to clone a large sequence in the reintegration plasmid so if this is an issue, try to use gRNAs closer together to reduce the length of sequence needing to be replaced. Additionally, HAs can be shortened (Beumer et al., 2013; Kanca et al., 2019). If cloning is problematic, the DNA can be commercially synthesized.

### Problem 3

No CRISPR positive flies.

### Potential solution

There are many possibilities for why CRISPR-Cas9 genome engineering is unsuccessful that relate to low viability of the flies or poor efficiency of editing. The DNA quality of plasmids for injection is crucial to ensure good viability so the DNA should be repurified to ensure it is clean. More embryos can be injected to increase the chances of an editing event and there are a range of commercial injection services that have experience in generating CRISPR edited flies. Post injection, dehydration of the vial containing survivors must be avoided as it can kill the larvae. Incubate the fly food vial containing injection survivors in a humidified incubator and add saturated filter paper to the fly food if it appears to be drying out. Adding liquid yeast paste to the food also increases survival of larvae. Finally, survival of adults is enhanced by removing them from the tube as soon as they eclose. The choice of line for injection also influences survival. Many researchers favor lines with *nos-Cas9* instead of *vas-Cas9*, as *nos* expression is more tightly confined to the germline (Port et al., 2015). Weak somatic *vas-Cas9* expression may lead to some lethality caused by somatic CRISPR events. In addition, if the locus being targeted is located on the X chromosome, it may be necessary to inject a greater number of embryos to achieve a successful modification. This is particularly relevant to the two-step protocol as it involves deletion of some sequences, which may cause lethality in males.

For the two-step CRISPR approach, try to reduce the amount of sequence deleted, as the efficiency of CRISPR-Cas9 events may depend on the size of the deletion. Even though large deletions of up to 30 kb have been successfully replaced with an attP site, smaller deletions can be obtained with a higher efficiency and limit rearrangements at the edited region (Poernbacher et al., 2019).

The one-step protocol describes crossing the injected flies to a stock carrying balancers, as this saves time. As balancer stocks can be less healthy, to maximize progeny and increase the chances of identifying an edited fly it may be better in some cases to first cross injection survivors to wildtype flies.

### Problem 4

No transformants are obtained for reintegration.

### Potential solution

Transformation efficiency with the ϕC31 system is usually very high. However, if no transformants are identified, make sure that the attP site was inserted into the genome correctly by sequencing, and that the attB site in the reintegration plasmid is intact. Sequence the ϕC31 integrase containing plasmid and ensure good DNA quality of all plasmids before injecting more embryos. The efficiency of reintegration is reduced when longer sequences are reintegrated.

## Resource availability

### Lead contact

Further information and requests for resources and reagents should be directed to and will be fulfilled by the lead contact, Hilary L. Ashe (hilary.ashe@manchester.ac.uk).

### Materials availability

Plasmids used in this study are available at Addgene or the *Drosophila* Genomics Resource Center. The RIV^cherry^+24xMS2-stem-loop plasmid and the *y*^*1*^ *w*^*67c23*^; *sna*^*Sco*^*/CyO* and the *yw, nos-Cas9* fly lines are available upon request from the lead contact. Other fly stocks are available from the Bloomington stock center.

### Data and code availability

This study did not generate or analyze datasets or code.
